# The Week's Work

**Published:** 1910-10-15

**Authors:** 


### The Week's Work.

### ROYAL HOSPITALS.

The Home Secretary has very properly drawn the attention of the Committee of the Portsmouth, Portsea and Gosport Hospital to the fact that the institution has at present no right to style itself "The Royal Portsmouth and Gosport Hospital." This intimation from headquarters has occasioned a great deal of feeling, we are informed, among members of the committee and supporters of the hospital, who are evidently under the impression that they are being unfairly singled out for reprimand. They point out that almost since its establishment in 1849 their institution has had the Royal patronage, and they maintain, if not in so many words then at least by inference, that they have therefore some sort of prescriptive right to the prefix Royal. A moment's reflection will show hospital workers how unfounded this assumption is. A charitable institution, like a corporation or society, is only granted the right to prefix the adjective Royal to its title by special permission from the Crown. In the case of hospitals such permission has always implied and expressed Royal approval of the good work done by the institution in question: it has been, as it were, the hallmark of excellence and as such an asset of very considerable value to the hospital. There is no comparison between such an institution and private houses which—we believe utterly illegally—style themselves Royal. There exists an old legal provision against the usurpation of the rights and privileges conferred by the prefix Royal, and although it will doubtless never be enforced in the case of private institutions, it is only just that the greatest care should be taken in limiting the term Royal, in the case of charitable institutions where the use of such a prefix may considerably influence public support, to those institutions which are actually under the Royal patronage. There are a few hospitals in London—the so-called "Royal hospitals"—which do possess the prescriptive right to the title, by virtue of charter, but so far as we are aware their position is unique, and certainly no precedent for such claims as are advanced from Portsmouth. If the Committee of the Portsmouth, Portsea and Gosport Hospital desires to retain the right to style its institution a Royal hospital, it should lose no time in petitioning the King for permisson to put his name at the head of the list of patrons and for special permission to express his approval of the work that the institution is doing by prefixing the word Royal to the name of the hospital.

### THE JEWISH HOSPITAL SCHEME.

We are glad to state that the resolutions of the recent meeting at which a decision was arrived at to proceed with the establishment of a Jewish Hospital in London have been subjected to keen criticism in the Jewish Press. The *Jewish World* points out that it is quite erroneous to regard the hospital scheme as one that is generally supported by the Jewish community. "The Jewish Hospital scheme is an old one, which is endeavouring to cover its past sluggishness and to take a new lease of life by declaring that the institution is to be erected as a memorial to the late King. It is a movement which has nothing whatever to do with the Sixpenny Fund. The latter, it may be recalled, was inaugurated at the end of May, shortly after the lamented death of King Edward, at a meeting at the Jewish Working Men's Club by a resolution proposed by the Chief Rabbi. In his speech Dr. Adler expressed the sympathy of Lord Rothschild with the project, and added that his lordship's suggestion was that in due course King George should be approached and his wishes consulted in the matter. At an early meeting of the committee of the fund it was decided that the memorial should *not* take the form of a hospital, and from the outset subscriptions were forwarded, and accepted, on that distinct understanding. Most of those contributing substantial sums made it quite plain that they were entirely opposed to a Jewish hospital. Following this, the Jewish Hospital Association decided to proceed with its three-year-old scheme and call it a memorial institution. This was evidently intended to force the hands of those members of the community who object to a hospital. The reply, however, was prompt and emphatic. Lord Rothschild and his brother, Mr. Leopold de Rothschild, sent a magnificent donation of two hundred guineas to the Sixpenny Fund, and the committee reaffirmed its decision that the memorial should not be a hospital." It is just as well that these explanations should be given wide publicity, in view of the fact that hospitals to commemorate some person or event usually succeed in winning a large share of support owing to the prevalence of what someone has called, with some show of justice, "the rampant philanthropy of the age." It is always difficult and somewhat invidious to point out to well-meaning but thoughtless persons the objections to a hospital scheme. In this case, however, the objections are so apparent that they need not be dilated upon. From a purely philanthropic point of view, we believe most Jews will agree that the establishment of such a special hospital will be a serious mistake. It is to be hoped that the scheme will be dropped as soon as possible in favour of the alternative memorial which has met with such general approval and support.

### THE FIRE AT LEEDS INFIRMARY.

The interesting fact about the fire at Leeds, of which we give an account on p. 91, is not that no one lost his life, but that no one lost his head. We may be quite sure that the order and intelligence which prevailed were no accident: discipline in the face of danger never comes by chance. It would be interesting to know what practice the staff at Leeds has had by way of preparation. How often are fire-drills held there? To what extent does each member of the staff have his own function in case of alarm? This last appears to be one of the most important points in good discipline, and was brought out forcibly in the article, called "Fire in Hospitals," which we published on December 18, 1909 (p. 336). The Secretary at Leeds Infirmary would be doing a very useful work if he published any lessons that the fire has brought home. What rules were proved valuable, what were a mistake, for bad rules as well as buildings perish in a fire? Another point, however, is worth notice, so obvious that it is forgotten by everybody: It is no use organising your staff unless you have a ready supply of water. At Leeds considerable time was lost, and therefore considerable damage done which might have been avoided, "owing to a difficulty in getting water." People, not only at Leeds, are apt to assume a supply as in the nature of things. The form of this difficulty should be made known so that other institutions may have the opportunity of guarding against a danger which, without the present instance, we should all say could not exist. If we must have fires at hospitals at all, let us not have them for nothing; the lesson Leeds has taught us must not be thrown away.

### THIS WEEK'S DRUG MARKET.

Compared with the unusually large number of price changes which took place last week, there are few alterations to record in the present report. Business continues to be fairly good and a considerable improvement upon the condition which was prevailing until a few weeks ago. English camphor refiners have reduced their prices by one penny per lb., mainly as a result of foreign competition. Buchu leaves are again very considerably cheaper, and the price of the variety most in request is about fifty per cent. below that quoted a month or so ago. Ipecacuanha is also slightly cheaper. Oil of cubebs is slightly lower in price, and a small reduction in the price of oil of almonds is expected. Hydrastis is again rather dearer, as also are coca-leaves. Oil of aniseed is fetching good prices. Sarsaparilla is rather dearer. Calumba-root is slightly cheaper. It is not improbable that the price of sulphonal may be advanced. Opium, morphine, codeine, cocaine, bromine salts, and mercurial salts are unchanged.

